# Modelling osteomyelitis

**DOI:** 10.1186/1471-2105-13-S14-S12

**Published:** 2012-09-07

**Authors:** Pietro Liò, Nicola Paoletti, Mohammad Ali Moni, Kathryn Atwell, Emanuela Merelli, Marco Viceconti

**Affiliations:** 1 Computer Laboratory, Cambridge University, William Gates Building, 15 JJ Thomson Avenue, Cambridge CB3 0FD, UK; 2 School of Science and Technology, Computer Science Division, University of Camerino, Via Madonna delle Carceri 9, Camerino (MC) 62019, Italy; 3 Department of Mechanical Engineering, University of Sheffield, Sir Frederick Mappin Building, Mappin Street, Sheffield S1 3JD, UK

**Keywords:** Osteomyelitis, osteoporosis, bone remodelling, RANKL, RANK, OPG, NF-kB, *Staphylococcus aureus*.

## Abstract

**Background:**

This work focuses on the computational modelling of osteomyelitis, a bone pathology caused by bacteria infection (mostly *Staphylococcus aureus*). The infection alters the RANK/RANKL/OPG signalling dynamics that regulates osteoblasts and osteoclasts behaviour in bone remodelling, i.e. the resorption and mineralization activity. The infection rapidly leads to severe bone loss, necrosis of the affected portion, and it may even spread to other parts of the body. On the other hand, osteoporosis is not a bacterial infection but similarly is a defective bone pathology arising due to imbalances in the RANK/RANKL/OPG molecular pathway, and due to the progressive weakening of bone structure.

**Results:**

Since both osteoporosis and osteomyelitis cause loss of bone mass, we focused on comparing the dynamics of these diseases by means of computational models. Firstly, we performed meta-analysis on a gene expression data of normal, osteoporotic and osteomyelitis bone conditions. We mainly focused on RANKL/OPG signalling, the TNF and TNF receptor superfamilies and the NF-*k*B pathway. Using information from the gene expression data we estimated parameters for a novel model of osteoporosis and of osteomyelitis. Our models could be seen as a hybrid ODE and probabilistic verification modelling framework which aims at investigating the dynamics of the effects of the infection in bone remodelling. Finally we discuss different diagnostic estimators defined by formal verification techniques, in order to assess different bone pathologies (osteopenia, osteoporosis and osteomyelitis) in an effective way.

**Conclusions:**

We present a modeling framework able to reproduce aspects of the different bone remodeling defective dynamics of osteomyelitis and osteoporosis. We report that the verification-based estimators are meaningful in the light of a feed forward between computational medicine and clinical bioinformatics.

## Background

There are two main types of bone tissues: *cortical bone*, and *trabecular bone*. The former is a compact tissue that makes up the outer shell of bones. It consists of a very hard (virtually solid) mass of bony tissue arranged in concentric layers called Haversian systems. Trabecular (also known as cancellous or "spongy") tissue is located beneath the compact bone and consists of a meshwork of bony bars (trabeculae) with many interconnecting spaces containing bone marrow. Both bone tissues undergo a continuous remodelling dynamics where old bone is replaced by new tissue ensuring the mechanical integrity and the morphology of the bone [1,2]. However, pathological conditions such as cancer, infection and autoimmune diseases can alter the equilibrium between bone resorption and bone formation, reducing bone density and increasing the risk of spontaneous fractures.

*Bone remodelling (BR) *is a cellular process conducted by *osteoclasts*, the cells responsible for bone resorption and by *osteoblasts*, the cells responsible for bone formation. Osteoblasts follow osteoclasts in a highly coordinated manner, forming the so-called *Basic Multi-cellular Units (BMUs)*. While osteoblasts and osteoclasts are located in the fluid part of the BMU, another type of cells, the *osteocytes*, are trapped in the bone matrix and they play a relevant role in the remodelling process. Osteocytes serve as mechanosensors: they translate mechanical stimuli at the tissue level into biochemical signals that flow through the osteocytic canalicular network to the BMU cells. In normal bone, the number of BMUs, the bone resorption rate, and the bone formation rate are all relatively constant [3].

**Figure 1:**
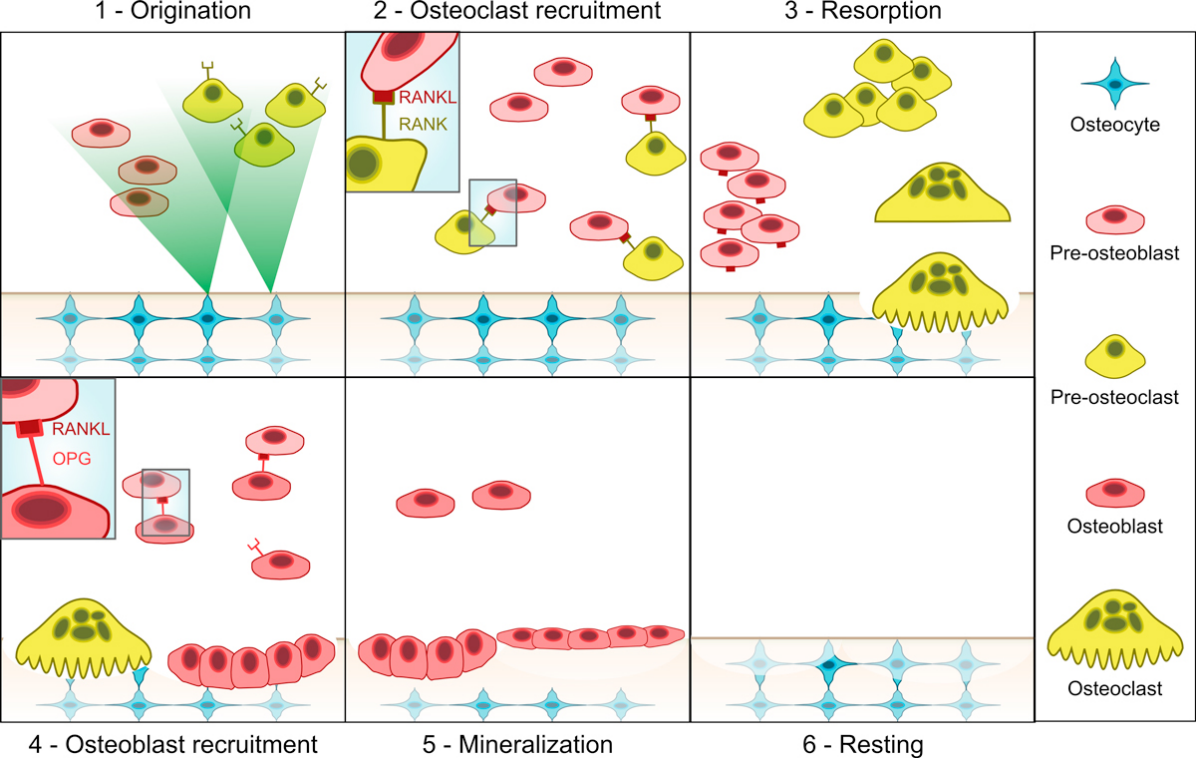
**Key steps in bone remodelling**. 1) Osteocytes send signals to the fluid part, activating pre-osteoblasts (*Pb*) and pre-osteoclasts (*Pc*). 2) *Pb*s express RANKL and *Pc*s express the RANK receptor. 3) RANK/RANKL binding induces *Pc*s' proliferation. *Pc*s enlarge and fuse, forming mature osteoclasts which start the bone resorption process. 4) Mature osteoblasts express the decoy receptor OPG and start the bone formation process. RANKL/OPG binding inhibits RANKL, thus protecting bone from excessive resorption. 5) During the mineralization process, osteoids secreted by osteoblasts calcify. 6) Finally in the resting phase, the initial situation is re-established.

The *RANK/RANKL/OPG *signalling pathway plays an important role in bone metabolism. RANK is a protein expressed by osteoclasts; RANK is a receptor for RANKL, a protein produced by osteoblasts. RANK/RANKL signalling triggers osteoclast differentiation, proliferation and activation, thus it prominently affects the resorption phase during bone remodelling. Osteoprotegerin (OPG) is a decoy receptor for RANKL. It is expressed by mature osteoblasts and it binds to RANKL, thus inhibiting the production of osteoclasts. Figure 1 shows the key steps during the bone remodelling process, that are:

1. **Origination**. During normal turnover or after a micro-crack, or as a response to mechanical stress, the osteocytes in the bone matrix produce biochemical signals showing sufferance towards the lining cells, i.e. the surface cells around the bone. The lining cells pull away from the bone matrix, forming a canopy which merges with the blood vessels.

2. **Osteoclast recruitment**. Stromal cells divide and differentiate into osteoblasts precursors. Pre-osteoblasts start to express RANKL, inducing the differentiation of and attracting pre-osteoclasts, which have RANK receptors on their surfaces. RANKL is a homotrimeric molecule displayed on the membrane of osteoblasts that stimulates differentiation in osteoclasts and is a key induction molecule involved in bone resorption leading to bone destruction.

3. **Resorption**. The pre-osteoclasts enlarge and fuse into mature osteoclasts. In cortical BMUs, osteoclasts excavate cylindrical tunnels in the predominant loading direction of the bone, while in trabecular bone they act at the bone surface, digging a trench rather than a tunnel. After the resorption process has terminated, osteoclasts undergo apoptosis.

4. **Osteoblast recruitment**. Pre-osteoblasts mature into osteoblasts and start producing osteoprotegerin (OPG). OPG inhibits the osteoclastic activity by binding to RANKL and preventing it from binding to RANK. When RANKL expression is high, osteoprotegerin levels are low and vice versa.

5. **Mineralization**. Osteoblasts fill the cavity by secreting layers of osteoids. Once the complete mineralization of the renewed tissue is reached, some osteoblasts can go apoptosis, other can turn into lining cells, while other can remain trapped in the bone matrix and become osteocytes.

6. **Resting**. Once the cavity has been filled by osteoblasts, the initial situation is re-established.

The bone remodelling undergoes a pathological process, generally related to ageing, termed osteopenia and with more severity, osteoporosis, during which an unbalance of the RANKL/OPG signalling equilibrium is typically observed. The osteoporosis is a skeletal disease characterized by low *Bone Mineral Density (BMD) *and structural fragility, which consequently leads to frequent micro-damages and spontaneous fractures; it is a chronic disease requiring long-term treatment. This disease primarily affects middle-aged women and elderly people and at present its social and economic impact is dramatically increasing, so much that the World Health Organization considers it to be the second-leading healthcare problem. While under normal circumstances, the ratio of RANKL/OPG is carefully balanced, the increase of RANKL plays an essential role in favouring resorption through osteoclast formation, function, and survival. With ageing and after a large number of remodelling cycles, the density of osteons increases and the cortical porosity and architectural defects of the bone increase as well. This leads to a vicious cycle where microdamages and consequently remodelling occur more and more frequently, weakening the bone structure and increasing the rate of spontaneous fractures [4]. Moreover, recent studies suggest that plasma levels OPG and RANKL are inversely related to bone mineral density and contribute to the development of osteoporosis in postmenopausal women [5], and thalassemia-induced osteoporosis [6]. One of the most worrying events is the infection of the bone which causes a disease called osteomyelitis. Similarly to osteoporosis, it is characterized by severe and rapid bone loss and by an unbalance at the molecular signalling level.

The aim of this work is to provide a computational modelling framework able to reproduce and compare the defective dynamics of osteoporosis and osteomyelitis. We believe that this framework could easily be adapted to model also other bone diseases like multiple myelomas or Paget's disease, and that it could help in better understanding the disruptions of cellular and signalling mechanisms that underlie such bone pathologies.

### Osteomyelitis

Osteomyelitis is a bone infection mainly caused by the aggressive pathogen *S. aureus*. Upon exposure to the bone, *S. aureus *induces a severe inflammatory response followed by progressive bone destruction and loss of the vasculature and with a persistent chronic infection; this is further complicated by the rapid emergence of resistant strains of *S. aureus*. Lab researches have shown that the infection prevents proliferation, induces apoptosis and inhibits mineralisation of cultured osteoblasts. The action of *S. aureus *increases RANKL expression and decreases OPG expression in osteoblasts in patients with staphylococcal osteomyelitis. Recent findings suggest that *S. aureus *SpA protein binds to osteoblasts, possibly through an interaction with the death receptor TNFR-1 which induces caspase 3 activation and apoptosis. The increase in RANKL is likely to trigger osteoclast-induced bone resorption and bone destruction and may help explain why patients with osteomyelitis have significant bone loss [7].

Although effective treatment of this disease is very difficult, one of most used drug is the fusidic acid that acts as a bacterial protein synthesis inhibitor by preventing the turnover of elongation factor G (EF-G) from the ribosome. Fusidic acid inhibits bacterial replication and does not kill the bacteria, and is therefore termed "bacteriostatic". Many strains of methicillin-resistant *S. aureus *(MRSA) remain sensitive to fusidic acid, but because there is a low genetic barrier to drug resistance (a single point mutation is all that is required), fusidic acid is usually combined with other antibiotics.

We believe that a model of the infection could provide a framework for a better diagnosis and understanding the antibiotic intervention. Here we develop a hybrid modelling framework for combining and untangling the relationships of physiological and molecular data. We then apply the methodology to determine disease related abnormalities of the key osteogenesis molecular network. The universality of the approach is demonstrated by an integration of the modelling and diagnosis which resembles medical visits with blood testing for infection progress and bone mineralisation measurements along a period of time. Our perspective is that this approach would inch towards an automatized methodology for improving disease classification and diagnosis.

## Results and discussion

### Meta analysis of gene expression data

**Figure 2:**
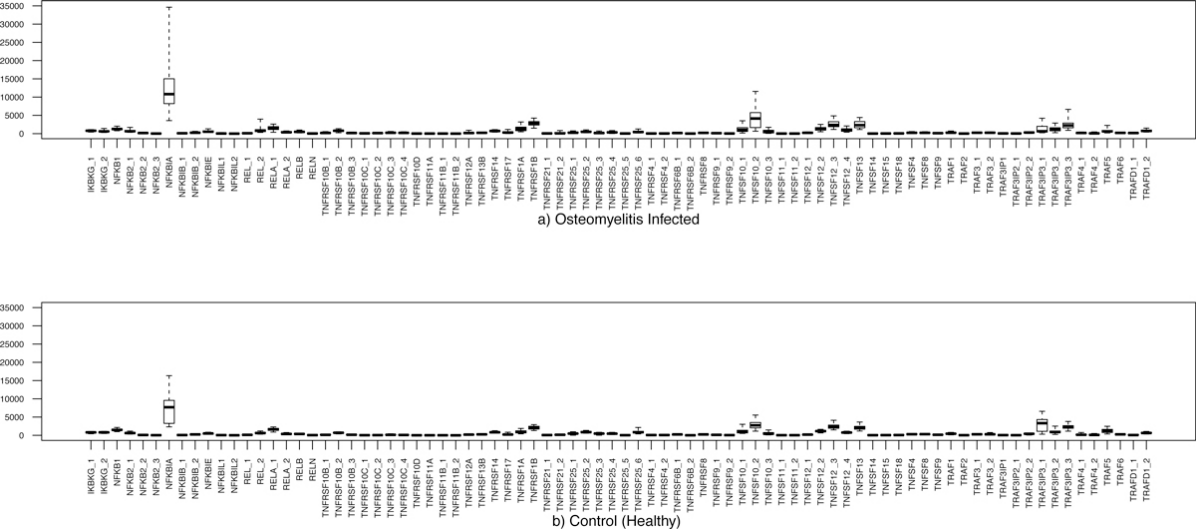
**Box plot representation of the gene expression of 82 genes corresponding to a) 48 osteomyelitis infected patients and b) 27 healthy controls)**.

**Figure 3:**
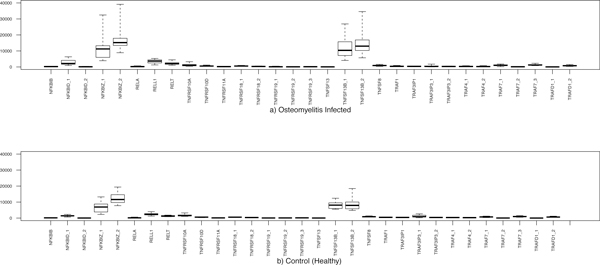
**Box plot representation of the gene expression of 31 genes corresponding to a) 43 osteomyelitis infected patients and b) 17 healthy controls)**.

Important parameter values of bone remodelling models are based on various authors (see [8] among others); here we also analysed more recent data, particularly available gene expression data. Since that both osteoporosis and osteomyelitis cause loss of bone mass, we decided to cross-compare gene expression datasets of both diseases. We have compared the expression levels of genes involved in osteomyelitis, osteoporosis patients and healthy controls using the box plots and comparison table (Figure 2, 3 and Table 1). We report in Table 1 the significant genes associated with the infection of osteomyelitis and or with the condition of osteoporosis. From the analysis of our data, we observe that few genes, related to TNF, TNF receptor superfamilies and to NF-kB have statistically different levels of expression in healthy controls, osteomyelitis and or osteoporosis. We observe that, with respect to control cases, for the microarray platform GPL96, 22 genes related to RANKL, RANK, OPG, NF-kB proteins, TNF and TNF receptor superfamilies are over expressed and 13 genes are down regulated in osteomyelitis (see Figure 2 and Table 1). There are other 47 genes that are weakly correlated with this infection (not shown). However, in case of GPL97 microarray platform, only 10 genes are highly expressed; 6 genes are down expressed (other 15 genes are weakly correlated in osteomyelitis) (see Figure 3 and Table 1). For the osteoporosis condition, using the platform GPL96, only 10 genes are up regulated and 6 are down regulated (see Table 1). It is notable in the platform GPL96, only 4 genes NFKB2_1, NFKB2_2, REL_2 and RELB are up-regulated in both types of diseases. In contrast, only 3 genes TNFRSF25_2, TRAF3IP3_1 and TRAF5 are down regulated in the both osteomyelitis infection and osteoporosis. However, 5 genes NFKB1, RELA_1, TNFRSF10B_2, TNFSF10_3 and TRAF3IP3_3 are differently regulated in osteomyelitis and osteoporosis.

**Table 1:** Comparative representation of gene expression level for osteomyelitis and osteoporosis.

Regulation for Osteomyelitis (GPL96)	Gene ID	Regulation for Osteomyelitis (GPL97)	Gene ID	Regulation for Osteoporosis (GPL96)	Gene ID
Up regulated	NFKB2_1	Up regulated	NFKB2_1	Up regulated	NFKB1
	NFKB2_2		NFKBIZ_1		NFKB2_1
	NFKBIA		NFKBIZ_2		NFKB2_2
	NFKBIE		RELL1		REL_2
	REL_2		RELT		RELA_1
	RELB		TNFSF13B_1		RELA_2
	TNFRSF10B_2		TNFSF13B_2		RELB
	TNFRSF10C_2		TRAF7_1		TNFRSF17
	TNFRSF10C _3		TRAF7_3		TNFSF10_2
	TNFRSF10C_4		TRAFD1_2		TRAF3_1
	TNFRSF1A	Down regulated	TNFRSF10A	Down regulated	TNFRSF10B_2
	TNFRSF1B		TNFRSF18_2		TNFRSF25_2
	TNFSF10_1		TRAF1		TNFSF10_3
	TNFSF10_2		TRAF3IP1		TRAF3IP3_1
	TNFSF10_3		TRAF3IP3_1		TRAF3IP3_3
	TNFSF12_3		TRAF3IP3_2		TRAF5
	TNFSF12_4				
	TNFSF12_2				
	TNFSF13				
	TRAF3IP3_2				
	TRAF3IP3_3				
	TRAFD1_2				
Down regulated	IKBKG 2				
	NFKB1				
	RELA_1				
	TNFRSF14				
	TNFRSF25_1				
	TNFRSF25_2				
	TNFRSF25_3				
	TNFRSF25_4				
	TNFRSF25_6				
	TRAF1				
	TRAF3IP2_2				
	TRAF3IP3_1				
	TRAF5				

Interestingly we found that, despite a very small increase of RANKL gene expression in osteoporosis and a larger increase in osteomyelitis, OPG gene expression become more deregulated in both osteomyelitis and osteoporosis. There is the increased expression of different isoforms of OPG which are known to have different binding capability with RANKL and seem to be linked, from mice experiments, to hypocalcemia [9]. Therefore we report that gene expression in osteoporosis and osteomyelitis could generate an unbalance between RANKL and OPG due to the different OPG isoforms, but also other genes, related to TNF, TNF receptor superfamilies and to NF-kB may be involved. Although gene expression and actual protein abundance are only loosely correlated, taking into account the results of gene expression data, we modified the autocrine and paracrine parameters of the existing mathematical model based on Komarova model [10]. We considered more appropriate to incorporate into the model the algebraic relationship of positive and negative regulators (such as RANKL and OPG) than just the RANKL change. On the basis of this consideration we developed new models for reproducing osteoporotic and osteomyelitis conditions.

### A computational framework for bone dynamics

In this work we present a combined computational framework for the modelling, simulation and verification of the bone remodelling process, and of bone pathologies like osteomyelitis and osteoporosis. Based on the methods developed in [11,12], this approach consists of the following two building blocks:

#### Mathematical model

We develop a differential equation model for describing the dynamics of bone remodelling and of bone-related pathologies at a multicellular level. The model describes the continuous changes of, and the interactions between populations of osteoclasts and osteoblast (including bacteria in the osteomyelitis model). Bone density is calculated as the difference between the formation activity which is proportional to osteoblasts concentration, and the resorption activity which is proportional to osteoclasts concentration. In the last twenty years, a variety of mathematical and computational models has been proposed in order to better understand the dynamics of bone remodelling (reviewed in [13-15]). Three main categories of models can be distinguished: those focusing on the organ level, where bone is described as a continuum material only characterized by its density; on the biomechanical properties and on the microstructural information at the tissue level; and on the cellular level where the interactions occurring among the different types of bone cells are concerned. The latter category can also incorporate intracellular signalling pathways and mechanosensing mechanisms (i.e. the process by which mechanical stimuli are translated into cellular signals). Our cellular-level model is based on the work by Komarova et al [10], where they developed an important model for BR based on experimental results described in Parfitt's work [8] which has inspired many other similar models. In particular we extended it in order to explicitly simulate bone pathologies: osteoporosis is reproduced by including an ageing factor that decreases the death rates of cells and by including a factor that increases the RANKL expression; osteomyelitis is modelled by adding a state variable for bacteria that affects the autocrine and paracrine regulation factors of osteoblasts and osteoclasts, similarly to Ayati's model on bone myeloma [16]. Although several efforts have been made in developing mathematical model for osteomyelitis and osteoporosis, molecular data has been rarely considered so far, regardless the availability of different gene expression microarray data related to osteomyelitis and osteoporosis and based on only single microarray database. So, we have developed mathematical model and showed the comparative study of gene expression data from different databases of similar platform to find out the genes expression level related to the RANKL, RANK, OPG and NF-kB proteins, which are strongly related to the osteomyelitis and osteoporosis.

#### Model verification

We define a stochastic model for bone remodelling from the ODE specification, that allows us to analyse the random fluctuations and the discrete changes of bone density and bone cells. Given that randomness is an inherent feature of biological systems, whose components are naturally discrete, the stochastic approach could give useful insights on the bone remodelling process. Indeed, stochasticity plays a key role in bone remodelling, e.g. the fluctuations in molecular concentrations of RANKL and OPG produce changes in the chemotaxis (the process by which cells move toward attractant molecules) of osteoclasts and osteoblasts. This may affect for example the cell differentiation, number and arrival time, and consequently the whole remodelling process. Besides achieving a good fitting between the ODE model and the stochastic one, we employ *probabilistic model checking *techniques for deriving three different clinical estimators that enable to assess the expected bone density, the density change rate, and the variance of bone density. Model checking is a static technique for automatically search for a property (specified as a logical formula) to hold or not over a definite set of states, and relies on qualitative properties: given a model and a property to verify, it returns an affirmative or a negative answer, i.e. the property holds or not. Differently, probabilistic model checking is equipped with quantitative information, and given a stochastic model and a property to verify, it returns the probability of the formula being satisfied. We believe that this kind of quantitative, formal and automated analysis may represent a step ahead in the understanding of bone diseases like osteomyelitis and osteoporosis, by shifting the attention from an informative, but empirical, analysis of the graphs produced by simulations towards more precise quantitative interpretations.

### Modelling bone remodelling pathologies

The ODE model for bone remodelling is mainly inspired from the work by Komarova et al [10], and describes the dynamics of osteoblasts' (*Ob*) and osteoclasts' (*Oc*) population in a BMU, and calculates the bone density as a function of *Ob *and *Oc *with the following equations:

$$\begin{array}{c} \frac{dO_{c}}{dt}=\alpha_{1}O_{c}^{g_{11}}O_{b}^{g_{21}}-\beta_{\text{1}}O_{c},\\ \frac{dO_{b}}{dt}=\alpha_{2}O_{c}^{g_{12}}O_{b}^{g_{22}}-\beta_{2}O_{b}. \end{array}$$

The model describes the autocrine and paracrine relationships between osteoclasts and osteoblasts. Autocrine signalling usually occurs by a secreted chemical interacting with receptors on the surface of the same cell. In the paracrine process a chemical signals that diffuse outside the emitting cell and interacts with receptors on nearby cells. Here the parameters *g_ij _*describe the effectiveness of autocrine and paracrine regulation, s.t. *g*_11 _describes the osteoclast autocrine regulation, *g*_22 _the osteoblast autocrine regulation, *g*_21 _is the osteoblast-derived paracrine regulation, and *g*_12 _is the osteoclast paracrine regulation. The nonlinearities of these equations are approximations for the interactions of the osteoclast and osteoblast populations in the proliferation terms of the equations. The autocrine signalling has a positive feedback on osteoclast production (*g*_11 _*>*0), and paracrine signalling has a negative feedback on osteoclast production (*g*_21 _*<*0). The autocrine signalling has a positive feedback on osteoblast production (*g*_22 _*>*0), and paracrine signalling has a positive feedback on osteoblast production (*g*_12 _*>*0).

Overall the regulatory circuit should lead to a positive mineralisation balance (*z*) which could be described by the expression $\frac{dz}{dt}=-k_{1}O_{c}+k_{2}O_{b}$ where *k*_1 _and *k*_2 _are the resorption and formation rates, respectively. More precisely, the bone density is determined by the difference between the actual resorption and formation activity when osteoclasts and osteoblasts exceed their steady levels. Therefore bone density is calculated as follows:

$$\frac{dz}{dt}=-k_{1}max\left(O_{c}-{Ō}_{c},0\right)+k_{2}max\left(O_{b}-{Ō}_{b},0\right),$$

where ${Ō}_{c}$ and ${Ō}_{b}$ denote the steady states of *O_c _*and *O_b_*, resp. For the spongy type bone we consider the variable *z *as the localized trabecular mass beneath a point on the bone surface.

In order to reproduce the defective dynamics (i.e. bone negative balance) characterizing osteoporosis, we assumed an increased death rate for osteoclasts and osteoblasts, motivated by the fact that the occurrence of defective bone pathologies in elderly patients is partly attributable to the reduced cellular activity typical of those patients. Therefore we introduced the parameter g_ageing _as a factor multiplying the death rates β*_i_*.

On the other hand, we modified the regulation factors in order to model an increased RANKL expression by osteoblasts, which results both from the analysis performed on gene expression data and from experimental evidences [6]. In our model *g*_21 _is the result of all the factors produced by osteoblasts that activates osteoclasts and as explained in [10], *g*_21 _= *RANKL OPG *where *RANKL *is the effectiveness of RANKL signalling while *OPG *is the effectiveness of OPG signalling. Therefore a further parameter g_por _has been included as a factor incrementing *g*_21_, in order to incorporate the changes in the system RANKL, OPG associated to osteoporosis. The resulting equations for osteoclasts and osteoblasts are:

$$\begin{array}{c} \frac{dO_{c}}{dt}=\alpha_{1}O_{c}^{g_{11}}O_{b}^{g_{21}+{\text{g}}_{\text{por}}}-{\text{g}}_{\text{ageing}}\beta_{1}O_{c},\\ \frac{dO_{b}}{dt}=\alpha_{2}O_{c}^{g_{12}}O_{b}^{g_{22}}-{\text{g}}_{\text{ageing}}\beta_{2}O_{b}. \end{array}$$

### Osteomyelitis effects on bone remodelling

Starting from the above model of bone remodelling, we consider the progressing of osteomyelitis induced by the *S. aureus *(variable *B*). Since several evidences show that the dynamics of the bacterial population follows a Gompertz curve, we consider an equation of the form

$$\frac{dB}{dt}=\gamma_{B}B\cdot log\left(\frac{s}{B}\right),$$

where γ*_B _*is the growth rate of bacteria, and *s *is the carrying capacity, i.e. the maximum population size. Additionally, we introduced four parameters *f_ij _*used to model the effects of the infection on the autocrine and paracrine regulation factors *g_ij_*. The resulting equations are:

$$\begin{array}{c} \frac{dO_{c}}{dt}=\alpha_{1}O_{c}^{g_{11}\left(1+f_{11}\frac{B}{s}\right)}O_{b}^{g_{21}\left(1+f_{21}\frac{B}{s}\right)}-\beta_{1}O_{c},\\ \frac{dO_{b}}{dt}=\alpha_{2}O_{c}^{g_{12}\left(1+f_{12}\frac{B}{s}\right)}O_{b}^{g_{22}-f_{22}\frac{B}{s})}-\beta_{2}O_{b},\\ \frac{dB}{dt}=\left(\gamma_{B}-V\right)B\cdot log\left(\frac{s}{B}\right). \end{array}$$

This model has been inspired from Ayati's work on multiple myeloma bone disease [16] and the key difference with respect to Komarova's model [10] is the addition of the terms *f_ij_B/s *that couple the bacterial density and its maximum size to the power laws for the osteoclast/osteoblast interactions. The bacterial parameters *f*_11_, *f*_12_, *f*_21_, *f*_22 _are all nonnegative. The *S. aureus*-induced infection affects the normal remodelling activity by:

• reducing osteoblasts' growth rate: in fact, the paracrine promotion of osteoblasts is reduced $\left(g_{12}/\left(1+f_{12}\frac{B}{s}\right)<g_{12},\;\text{since}\;g_{12}>0\right)$, and the autocrine promotion of osteoblasts is reduced as well $\left(g_{22}-f_{22}\frac{B}{s}<g_{22}\right)$;

• increasing RANKL and decreasing OPG expression: as previously stated, the paracrine inhibition of osteoclasts is a negative exponent resulting from the difference between the effectiveness of OPG signalling and that of RANKL signalling. Since $g_{21}\left(1-f_{21}\frac{B}{s}\right)>g_{21}$, the infection causes an increase in RANKL expression and therefore a decrease in OPG expression.

In addition the infection increases the autocrine promotion of osteoclasts (since *g*_11 _*>*0). We have taken γ*_B _*to be independent of bone loss. The parameter *V *describes the effectiveness of the antibiotic treatment as a factor decreasing the growth rate γ*_B _*of bacteria. Two different kinds of treatment can be distinguished: bacteriostatic treatments that stop bacteria proliferation (*V *= γ*_B_*); and bacteriocide treatments which kill bacteria (*V *> γ*_B_*).

**Figure 4:**
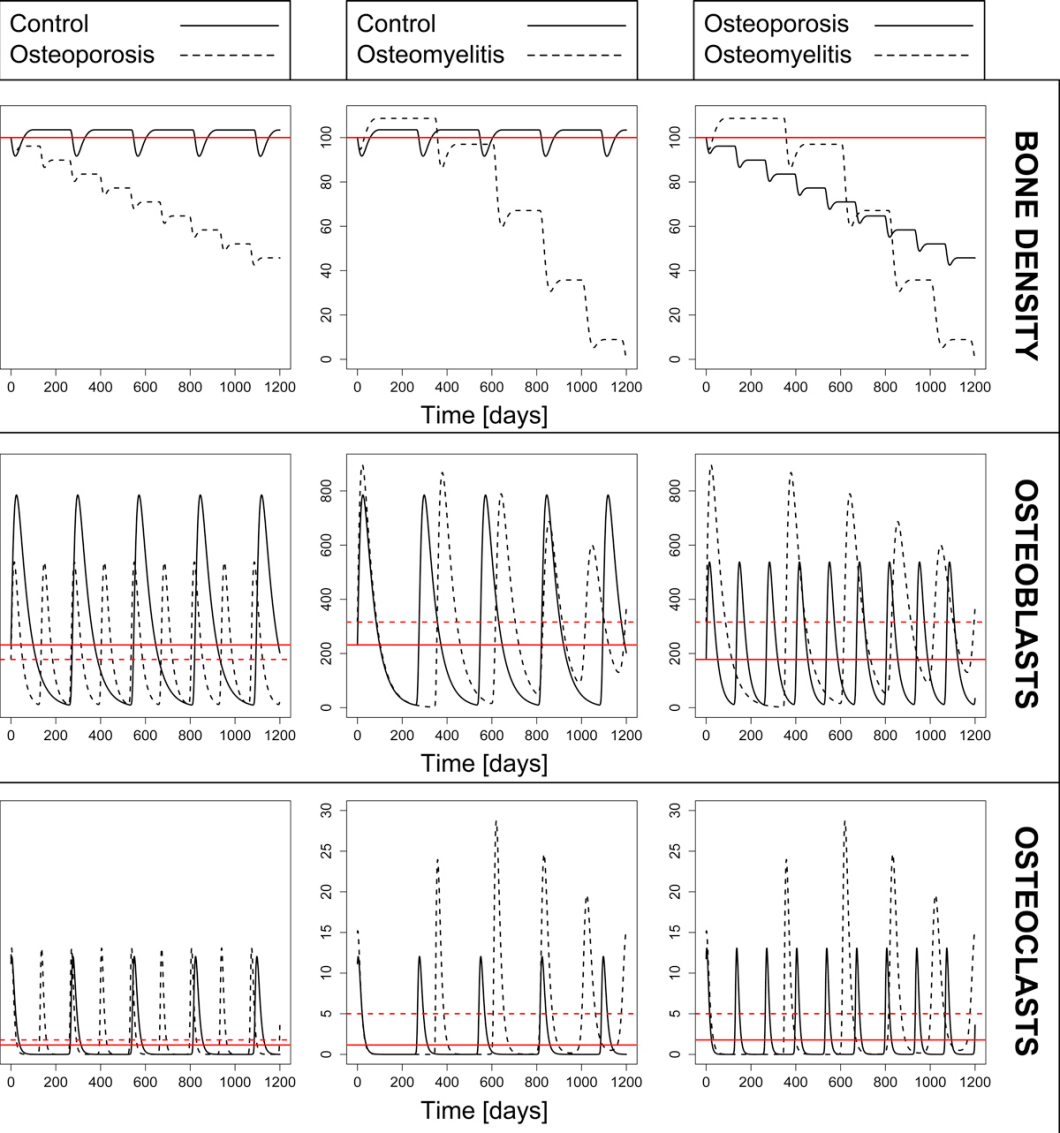
**Simulation results of the ODE model**. Bone density (first row), number of osteoblasts (second row), and number of osteoclasts (third row) compared between control and osteoporotic (first column); control and osteomyelitis (second column); osteoporotic and osteomyelitis (third column). Red lines mark the steady states for the variables considered. Results show a negative remodelling balance in the osteoporotic case and much more critical in the osteomyelitis case. While we observe a higher (but constant) remodelling rate in the osteoporotic configuration, in the osteomyelitis scenario the remodelling period is unstable and longer.

Parameters for the three different models (control, osteoporosis and osteomyelitis) are given in Table 2. Simulation results for bone density, osteoblasts and osteoblasts under the three different scenarios are compared in Figure 4. The plots show that both osteoporosis and osteomyelitis are characterized by a negative remodelling balance, but in the latter case the bone loss becomes much more critical after 600 days. In the osteoporotic case, the remodelling period is shorter than in the control case, mimicking the fact that in older patients microfractures and consequently remodelling occur more frequently, in a vicious cycle that progressively weakens the structure and density of the bone [4]. On the other hand, the regular cycles of the normal bone model above are perturbed by the presence of the infection (chronic), and we observe longer and unstable remodelling periods.

**Table 2:** Model parameters.

Parameter	Description	Value
(α_1_, α_2_)	*O_c _*and *O_b _*growth rates	(3, 4) *day*^-1^
(β_1_, β_1_)	*O_c _*and *O_b _*death rates	(0.2, 0.02) *day*^-1^
(*g*_11_, *g*_12_, *g*_22_, *g*_21_)	Effectiveness of autocrine/paracrine regulation	(1.1, 1, 0, -0.5)
(*k*_1_, *k*_2_)	Resorption and formation rates	(0.0748, 0.0006395) *day*^-1^
g_ageing_	Ageing factor	2
g_por_	RANKL factor	0.1
γ*_B_*	*S. aureus *growth rate	0.005 *day*^-1^
*s*	*S. aureus *carrying capacity	100
*V*	Effectiveness of antibiotic treatment	(0.005, 0.007) *day*^-1^
*t_treat_*	Dosage time	(200, 400, 600) *days*
(*f*_11_, *f*_12_, *f*_22_, *f*_21_)	Effect of infection on regulation factors	(0.005, 0, 0.2, 0.005) *day*^-1^
$\left(\bar{O_{c}},\bar{O_{b}}\right)$	Steady levels of *O_c _*and *O_b_*	Control: (1.16, 231.72)
		Osteoporosis: (1.78, 177.91)
		Osteomyelitis: (5, 316)
(*O*_*c*0_, *O*_*b*0_, *B*_0_)	Initial states	Control: (11.16, 231.72, 1)
		Osteoporosis: (11.78, 177.91, 1)
		Osteomyelitis: (15, 316, 1)

Values have been adapted from literature (mainly [10,16]). Some parameters are specific to a particular scenario: g_ageing _and g_por _are relative to the osteoporosis model, while parameters γ*_B_, s, V, t_treat _*and (*f*_11_, *f*_12_, *f*_22_, *f*_21_) are specific to the osteomyelitis model.

**Figure 5:**
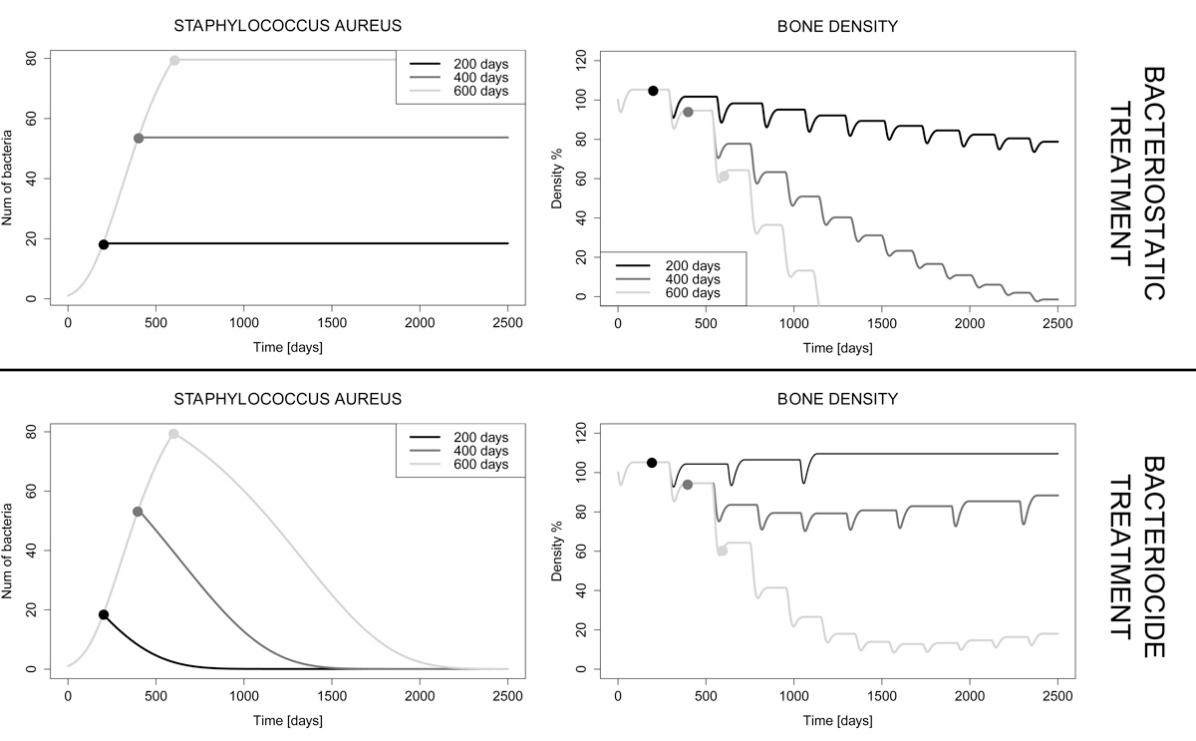
**Simulation of a bacteriostatic (*V *= 0.005 = γ*_B_*) and a bacteriocide (*V *= 0.007 *>*γ*_B_*) treatment for *S. Aureus *at different dosage times (200, 400 and 600 days)**. Dots on the plots mark the points when treatment is given. As regards the bacteriostatic drug, the bone density is not subject to critical drops if the treatment is administered at *t *= 200 days; in the other cases, the normal remodelling activity cannot be re-established, even if the bone loss is less critical w.r.t. the untreated infection. On the other hand, the bacteriocide treatment looks more effective than the bacteriostatic one, although recovering the normal density becomes much more difficult if the drug is administered later than *t *= 400 days.

Furthermore we simulate the dosage of a bacteriostatic treatment (*V *= 0.005 = γ*_B_*) and of a bacteriocide treatment (*V *= 0.007 *>*γ*_B_*) for *S. Aureus *at different dosage times *t_treat _*= 200, 400 and 600 days. Figure 5 shows that when applying the bacteriostatic drug (e.g. fusidic acid), the severe bone loss characterizing osteomyelitis can be limited only if the treatment is administered at *t *= 200 days. With later dosages the normal remodelling activity cannot be re-established, even if the situation is considerably better w.r.t. an untreated infection. Conversely, the bacteriocide treatment looks more effective than the bacteriostatic one, and the bone activity is able to recover regardless the dosage time. However the recovery time becomes longer as *t_treat _*increases. Therefore in both antibiotic treatments timeliness is a crucial factor in order to effectively operate against the infection.

### Stochastic model for the verification of bone pathologies

Following and extending the work in [11], we define a stochastic model for bone remodelling and perform formal analysis by means of probabilistic verification techniques, which allow to assess the probability of a particular configuration of the biological system (usually expressed as a logical formula) being reached. In our settings we derive a *Continuous Time Markov Chain (CTMC) *from the mathematical model described above and we use the model checker PRISM [17].

We follow a *population-based *approach where a state of the system is determined by the discrete density of the different cell populations involved. Osteoclasts, osteoblasts and bacteria populations are specified as *PRISM modules*, consisting of a random state variable modelling the number of individuals; and with a list of stochastic transitions in a guarded-command syntax of the form

[label]guard→rate:update

where *label *is an optional transition label; *guard *is a predicate over the state variables determining whether a transition is enabled or not; in the CTMC settings, *rate *is the speed/propensity of the action, giving rise to an exponentially distributed duration of the transition with mean 1/*rate *(faster action have a higher probability of being taken than slower one); and *update *optionally sets new values to state variables. The ODE model is translated into a set of PRISM guarded commands by applying the following method [18]. Consider a simple ODE population model of the form $\frac{dx}{dt}=\alpha -\beta$. The corresponding PRISM transitions would be:

$$\begin{array}{c} x<x_{max}\to \alpha :x=x+1\\ x<x_{min}\to \beta :x=x-1 \end{array}$$

where *x_max _*and *x_min _*are the maximum and the minimum *x*, resp. In other words, growth rates in the ODE model become the stochastic rates in a transition incrementing the population size, while death rates are involved in the transitions decrementing the population size.

Table 3 summarizes the transitions of osteoclasts, osteoblasts and bacteria in the PRISM model. Moreover, in order to reduce the state-space of the stochastic model, bone density has not been implemented as a state variable, but by means of transition rewards, i.e. positive costs associated to transitions. We implement a couple (*boneFormed, boneResorbed*) of rewards associated to osteoblasts' and osteoclasts' transitions where their stochastic rate is the formation rate (*k*_2_*O_b_*) and the resorption rate (*k*_1_*O_c_*), respectively (see Table 3).

**Table 3:** Transitions in the stochastic model for bone remodelling.

(a) Osteoclasts			
[]	$0<O_{c}<O_{c_{max}}\Lambda \;O_{b}>0\to$	$\alpha_{1}O_{c}^{g_{11}\left(1+f_{11}\frac{B}{s}\right)}O_{b}^{g_{21}\left(1-f_{21}\frac{B}{s}\right)}$	: *O_c _*= *O_c _+ *1
[]	*O_c _*> 0 →	g_ageing_β_1_*O_c_*	: *O_c _*= *O_c _- *1
[*resorb*]	*O_c _*> 0 →	*k*_1_*O_c_*	: *true*

(b) Osteoblasts			

[]	$0<O_{b}<O_{b_{max}}\Lambda \;O_{c}>0\to$	$\alpha_{2}O_{c}^{g_{12}\left(1+f_{11}\frac{B}{s}\right)}O_{b}^{g_{22}-f_{22}\frac{B}{s})}$	: *O_b _*= *O_b _+ *1
[]	*O_b _*> 0 →	g_ageing _β_2_*O_b_*	: *O_b _*= *O_b _- *1
[*form*]	*O_b _*> 0 →	*k*_2_*O_b_*	: *true*

(c) Bacteria			

[]	0 <*B *<*B_max _*∧ *treat *= 0 →	$\gamma_{B}B\cdot log\left(\frac{s}{B}\right)$	: *B *= *B *+ 1
[]	*treat *= 0 →	$\frac{1}{t_{treat}}$	: *treat *= 1
[]	0 <*B *<*B_max _*∧ *treat *= 1 ∧ *V *< γ*_B _*→	$\left(\gamma_{B}-V\right)B\cdot log\left(\frac{s}{B}\right)$	: *B *= *B *+ 1
[]	*B *> 0 ∧ *treat *= 1 ∧ *V *> γ*_B _*→	$\left(V\;-\;\gamma_{B}\right)B\cdot log\left(\frac{s}{B}\right)$	: *B *= *B *- 1

(d) Bone resorbed reward		(e) Bone formed reward	
	
[*resorb*] *true*: 1		[*form*] *true*: 1	

We consider the model with bacterial infection, being equivalent to the model with no infection when *f_ij _*= 0. Guard predicates are set in order to avoid out-of-range updates and 0-valued transition rates. Maximum values for state variables have been estimated from the continuous model. The variable *treat *is used as a switch for the beginning of treatment firing with rate 1/*t_treat_*, therefore with an exponentially distributed delay having mean *treatTime*. Bacteriocide (*V >*γ*_B_*) and non-bacteriocide (*V <*γ*_B_*) dynamics is considered separately. Bone density is calculated by subtracting the bone resorbed reward (d) from the bone formed reward (e). Resorption and formation rates in the ODE model, i.e. *k*_1_*O_c _*and *k*_2_*O_b _*respectively, become the stochastic rates of transitions incrementing the bone resorbed/formed reward.

### Potentialities in clinical bioinformatics and conclusions

Osteomyelitis and osteoporosis are assessed through the verification of quantitative properties over the defined stochastic model.

Let assume that the simulation of the PRISM implementation of the model is run in parallel with the determination of clinical parameters during the periodic medical visits of a patient. These medical visits provide a mean of fine tuning a personalised model of the disease and a measure of how a therapy is effective. Different diseases, when monitored in a continuous way, may produce different alterations in local mineral density. We could extend the statistical estimators of a disease to: 1) the BMD (measured as z-score, the number of standard deviations above or below the mean for the patients age, sex and ethnicity; or as t-score, i.e. the number of standard deviations above or below the mean for a healthy 30 year old adult of the same sex and ethnicity as the patient); 2) The rate of change of BMD. This estimator tells us the emergence of defects of the bone metabolism in terms of signaling networks of RANK/RANKL and decrease of pre-osteoblast number; 3) The variance, skewness and curtosis of the the local small scale intermittency of the signal. For example osteomyelitis and osteoporosis show slightly confounding pattern of BMD decrease; we could also think at the confounding patterns of IRIS in HAART therapy, co-morbidity of osteopetrosis and osteoporosis, multiple myelomas, breast cancer, diabetes and metabolic syndromes, etc. The variance could perhaps help in discriminating among bone-related diseases. From a technical viewpoint, properties to verify have been formulated in *CSL (Continuous Stochastic Logic) *[19], and they give rise to three clinical estimators that we evaluate over 1200 days (about four years), which is enough to assess the presence of bone diseases:

• **Bone density estimator**. It is calculated as the difference between the cumulative $\left(C^{\leq t}\right)$ expected rewards (ℛ_{"..."}_) for bone formation and bone resorption, with the formula

$$f_{BD}\left(t\right):R_{\left\{\prime \prime \text{bone}\;\text{Formed}\prime \prime \right\}}=?\left[C^{\leq t}\right]-R_{\left\{\prime \prime \text{bone}\;\text{Resorbed}\prime \prime \right\}}=?\left[C^{\leq t}\right],\;t=0,\;10,...,1200$$

• **Density change rate**. It allows to assess rapid negative and positive changes in bone density. This estimator could be particularly helpful in detecting the insurgence of osteomyelitis before critical values of bone density are reached, since osteomyelitis is typically characterized by a higher negative change rate than osteoporosis. In particular the estimator is defined as the difference quotient of BMD over a time interval of months, e.g. 50 days. The formula obtained is

$$\frac{f_{BD}\left(t+\Delta t\right)-f_{BD}\left(t\right)}{\Delta t},\;t=0,10,...,1200.$$

• **Density variance**. While the first estimator computes the expected value of bone density, here we calculate the variance of BMD taking into account the whole state space and the actual bone density at each state.

**Figure 6:**
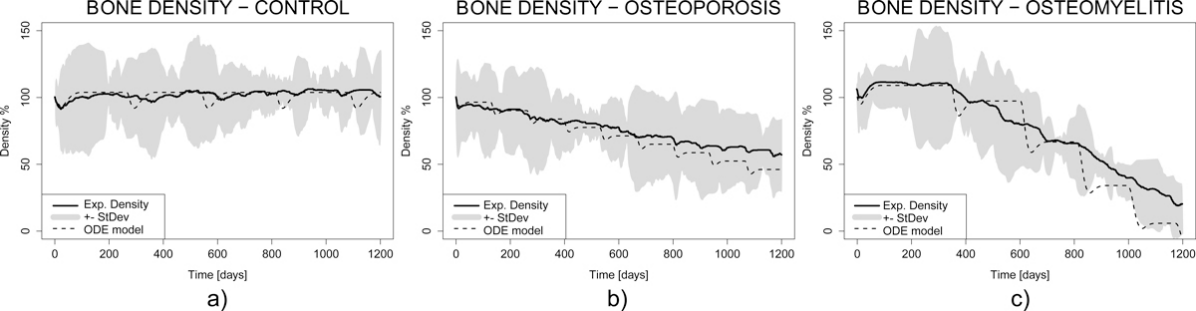
**Bone mineral density function and its standard deviation for the control (left, a), osteoporosis (middle, b) and osteomyelitis (right, c) simulations**.

**Figure 7:**
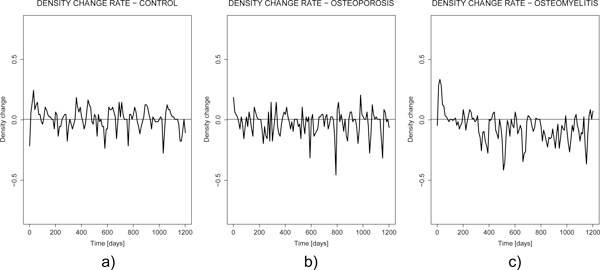
**Rate of change of bone mineral density function for the control (left, a), osteoporosis (middle, b) and osteomyelitis (right, c) simulations**.

Figure 6 and Figure 7 describe bone mineral density, standard deviation and density change rate functions for the control (a), osteoporosis (b) and osteomyelitis (c) cases, respectively. Clearly the osteomyelitis case shows quicker decrease than control and osteoporosis cases. They provide an example of how the diagnostic estimators could be derived. Therefore our work is meaningful in perspective of a clinical bioinformatics characterized by a close coupling between clinical measures and modelling prediction.

Here we report that the genetic complexity and the gene expression data meta analysis shows that the underlying "mystery" of bone remodelling is much greater than handled by the current mathematical models. In other words we are not able to use all our gene expression results in a full model of BR diseases. Although our model of osteomyelitis and the comparison with the osteoporosis is not able to consider all this complexity, nevertheless it makes a partial use of the results of the analysis of the experimental data and produces a realistic description of the pathology. From a methodological point of view the combination of mathematical and formal method approach has led to the proposal of considering additional estimators (first derivatives and variance) of the bone pathologies as diagnostic tool. That could also inspire the ideal situation in which a personalised model is generated from (personalised) data and the comparison between clinical data obtained during periodic medical check-up is compared with the computer predictions.

## Methods

### Data analysis

We found that there are no comprehensive analysis on osteomyelitis; most studies focus on specific conditions. We have collected a large ensemble of gene expression data related to osteomyelitis and osteoporosis. For this reason, we have considered 6 microarray data sets of the same platform GPL96 from the Gene Expression Omnibus (http://www.ncbi.nlm.nih.gov/geo/), accession numbers are GSE16129, GSE6269, GSE11907, GSE11908, GSE13850 and GSE7429 [20-23]. We observe that RANKL, RANK, OPG and NF-kB proteins impact more on the bone remodelling for osteomyelitis and osteoporosis [7,20-22]. For this reason to understand the effect osteomyelitis and osteoporosis on bone remodelling, we have considered the genes related to the proteins RANKL, RANK, OPG, NF-kB proteins, TNF and TNF receptor superfamilies. We observed that there are 82 genes are related with these proteins. So, we filtered the required 82 genes related data. We have selected samples for 48 infected and 27 healthy controls for osteomyelitis and 30 infected and 30 healthy controls for osteoporosis. The datasets contain data from people of different age and sex.

For more evidence about osteomyelitis, we have considered more gene expression data related to osteomyelitis on different platform GPL97. For this reason, we have considered additional 3 microarray data sets from the Gene Expression Omnibus (http://www.ncbi.nlm.nih.gov/geo/), accession numbers are GSE6269, GSE11907 and GSE11908 [21,22]. To understand the effect of osteomyelitis on the bone remodelling, we have considered the genes related to the proteins RANKL, RANK, OPG, NF-kB proteins, TNF and TNF receptor superfamilies like previous analysis. We observed that in the platform GPL97, there are 31 genes are related to these proteins and superfamilies. So, we filtered the required genes related data. We have selected samples for 43 infected and 17 healthy controls. Standard anova and Box plots representation were used to analyse and visualise the expression levels of these genes for the infection of osteomyelitis and osteoporosis condition. We output in Table 1 the groups of over expressed and under expressed categories.

### ODE and probabilistic model checking models

We have implemented the ODE model based on Komarova et al [10] in *R*, and using the *FME *package [24] to analyse parameter sensitivity and robustness. We have used *Mathematica *and *MATLAB *for steady states and ODE calculation using state of art numerical routines. Scripts and functions for the models could be made available upon request to the first author. For the specification of the stochastic model and for performing probabilistic verification we have adopted the open-source PRISM probabilistic model checker [17], one of the reference existing model checkers for the analysis of systems which exhibits random or probabilistic behaviour. Since model checking is based on graph-theoretical techniques for exploring the whole state space of the model, this task becomes computationally infeasible for non-trivial models, due to the combinatorial explosion of the state space. For this reason, verification has been performed by means of *approximate probabilistic model checking *techniques that calculate the probability of a given property on a statistical basis, i.e. by sampling on a number of simulations of the model. In this work we have taken 20 samples for each verified property, that were enough to reproduce outputs similar to the non-approximate verification. PRISM models could be made available upon request to the second author.

## Competing interests

The authors declare that they have no competing interests.

## Authors' contributions

LP and NP conceived and designed the models, MM carried out data analysis. All authors contributed writing, reading and approving the final manuscript.
